# Using biothesiometer, Neuropathy Symptom Score, and Neuropathy Disability Score for the early detection of peripheral neuropathy: A cross-sectional study

**DOI:** 10.5339/qmj.2024.24

**Published:** 2024-07-29

**Authors:** Ching Siew Mooi, Kai Wei Lee, Abdul Hanif Khan Yusof Khan, Navin Kumar Devaraj, Ai Theng Cheong, Fan Kee Hoo, Wan Aliaa Wan Sulaiman, Wei Chao Loh, Leong Yong Jian, Teh Xian Hui, Vasudevan Ramachandran

**Affiliations:** 1 Department of Family Medicine, Faculty of Medicine and Health Sciences, Universiti Putra Malaysia, Serdang, Selangor, Malaysia; 2 Malaysian Research Institute on Ageing, Faculty of Medicine and Health Sciences, Universiti Putra Malaysia, Petaling Jaya, Malaysia; 3 Department of Medical Sciences, School of Healthcare and Medical Sciences, Sunway University, Bandar Sunway, Selangor, Malaysia *Email: sm_ching@upm.edu.my; 4 Department of Medical Microbiology, Faculty of Medicine and Health Sciences, Universiti Putra Malaysia, Serdang, Selangor, Malaysia; 5 Department of Neurology, Faculty of Medicine and Health Sciences, Universiti Putra Malaysia, Serdang, Selangor, Malaysia; 6 Faculty of Health Sciences, University College MAIWP International, Taman Batu Muda, Batu Caves, Kuala Lumpur, Malaysia; 7 Department of Conservative Dentistry and Endodontics, Saveetha Dental College and Hospitals, Saveetha Institute of Medical and Technical Sciences, Chennai, Tamil Nadu, India

**Keywords:** Peripheral neuropathy, biothesiometer, sensitivity and specificity

## Abstract

Patients with peripheral neuropathy could have damaged peripheral nerves, which leads to sensory and motor dysfunction. Diabetes, infections, and trauma are the major causes of peripheral neuropathy. Vibratory perception threshold (VPT) tools are commonly used to detect peripheral neuropathy. This study aims to determine the assessment of peripheral neuropathy through the different diagnostic tools in the community in Malaysia. A total number of 1283 participants were recruited from the seven retail pharmacies located in Selangor, Malaysia. The peripheral neuropathy test was conducted based on VPT tools on both feet using the digital biothesiometer. Following that, Neurological Symptom Score (NSS) and Neurological Disability Score (NDS) were taken from the participants to assess the neurological symptoms. Participants had an average age of 40.6 ± 12.9 years and were mostly of Chinese ethnicity (54.1%). The findings show that increasing age was associated with more severe peripheral neuropathy across the various assessment tools, but gender differences were found with the biothesiometer test and ethnicity has severity in the biothesiometer and disability scores. The sensitivity and specificity of the biothesiometer test were 0.63 and 0.84, respectively. The combined tool NSS and NDS had high specificity and a high positive predictive value, suggesting that it could be a reliable indicator of peripheral neuropathy when both scores are elevated. The findings show that the biothesiometer test, NSS, and NDS are considered screening VPT tools for diagnosing peripheral neuropathy. However, further evaluation and diagnostic testing are necessary in cases of a positive test result.

## Introduction

Peripheral neuropathy is a condition that affects the peripheral nervous system and can present with a range of symptoms, some of which may be vague. This is a common complication of diabetes mellitus, which affects the peripheral nervous system and manifests in a diverse range of symptoms, some of which may be indistinct.^1-4^ Symptoms associated with diabetic peripheral neuropathy (DPN) may differ based on the specific nerves that are affected. Numbness, prickling, or tingling in the hands or feet may develop gradually as a result of sensory nerves receiving information from the skin, including temperature, pain, vibration, and contact. Muscle cramping, lethargy, or twitching may result from motor nerves that regulate muscle movement.^5-9^ The diagnosis of DPN, along with the assessment of its worldwide prevalence and incidence rates, continues to pose challenges. Diverse viewpoints exist regarding the efficacy of expanding screening efforts to facilitate early diagnosis and the initiation of treatment prior to the onset and progression of the condition.

Various screening tools have been developed to detect DPN, including the biothesiometer, Neuropathy Symptom Score (NSS), and Neuropathy Disability Score (NDS).^7-9^ However, the sensitivity, specificity, positive predictive value (PPV), and negative predictive value (NPV) of these tools have not been consistently reported in the literature. A study’s findings have shown that vibratory perception threshold (VPT) measured with biothesiometer exhibits favorable diagnostic accuracy in detecting DPN when compared with clinician diagnosis, neuropathy symptom scores, and abnormal nerve conduction.^5^ The Michigan Neuropathy Screening Instrument (MNSI) is a composite measure of vibration perception with a 128-Hz tuning fork, thermal perception with a metallic rod, pin-prick sensation, and Achilles tendon reflexes.^3^ The NSS is a simple and reliable tool that assesses the presence and severity of neuropathic symptoms.^10^ The NDS is a commonly used clinical examination method that assesses neuropathy signs.^4^

A study reported that 35% of patients with type 2 diabetes had peripheral neuropathy diagnosed with NSS and NDS.^11^ A study among patients with type 2 diabetes mellitus attending a follow-up visit in an outpatient clinic at Universiti Kebangsaan Malaysia Medical Center found that the prevalence of DPN was 79.1%.^12^ Another study in the Primary Care Clinic, Universiti Hospital, which included 138 diabetic patients assessed using the NSS and NDS, reported that the prevalence of DPN was high at 50.7%13 and another study conducted among the Malaysian population reported 54.1% of DPN based on nerve conduction study.^11^

While the biothesiometer, NSS, and NDS are commonly used screening tools for DPN, their diagnostic performance varies depending on the study population and diagnostic criteria used.^7-9^ The objectives of this study were as follows: to determine the sensitivity, specificity, PPV, and NPV of the biothesiometer test, NSS questionnaire, and NDS questionnaire for detecting peripheral neuropathy compared with the reference standard of clinical examination findings; to evaluate if the combined use of the NSS and NDS questionnaires with a threshold score >10 provides higher accuracy in diagnosing peripheral neuropathy compared with the individual scores; and to assess the association of age, gender, and ethnicity on peripheral neuropathy severity across the various screening modalities.

## Methods

### Study setting, study population, and sampling method

This cross-sectional study was conducted in a community setting between March 15, 2021, and May 5, 2022, at seven retail pharmacies in Selangor, Malaysia. This study was conducted in Selangor due to its high population size, making it the largest state in Malaysia to reflect real-world conditions and provide insights into the early detection of peripheral neuropathy in a non-clinical environment. The inclusion criteria for this study were that the participants must be Malaysian, aged ≥18 years, and willing to sign the informed consent form. Those critically ill and/or mentally challenged were not eligible to participate in this study. Participants were recruited using a convenient sampling method.

### Sample size calculation

Using the StatCalc feature in Epi Info 7.0, the sample size was determined by the prevalence of peripheral neuropathy, which was 2.38% among the Parsi community in Bombay.^14^ The sample size required was 899 with a 95% confidence interval (CI), power of 80%, and a p-value of <0.05. However, to account for potential incomplete or missing data at a rate of 30%, the total number of participants needed was adjusted to 1283.

### Data collection tools

The questionnaire is self-administered and clarified with the researchers. The questionnaire has been divided into four sections, each serving a unique purpose. In the initial section, we sought to collect comprehensive socio-demographic information such as age, gender, ethnicity, and personal monthly income in Ringgit Malaysia. In addition, this section probed participants about their lifestyle factors, including alcohol consumption, smoking habits, and dietary preferences (vegetarianism). Co-morbidities were explored in this section, with inquiries regarding the presence of conditions such as hypertension, diabetes, neurological disorders, and any family history of neurological disorders. Subsequent sections of the questionnaire were dedicated to the evaluation of peripheral neuropathy through screening tests, as well as the assessment of neuropathy symptom scores and neuropathy disability scores.

### Biothesiometer test

The peripheral neuropathy test was the determination of the VPT on both feet using the digital biothesiometer by P&G (Diabetik Foot Care Model: Vibrometer-VPT model 1; The Digital 0–50 V indicator with a portable Vibration probe functioning at 230 V, ±20%, AC, 50-Hz Mains operation). The biothesiometer probe can vibrate with an amplitude proportional to the square of the applied voltage. To test the vibration perception threshold, a vibration probe must be placed on six sites on each foot. The sites are the plantar aspects of the tip of the first toe, the base of the first, third, and fifth toes, the medial aspect of the midfoot and at the heel.

After patients were familiarized with the sensation by holding the probe against the distal palmar surface of the hand, the probe was then applied perpendicular to the distal plantar surface of the big toe of both legs. The voltage slowly increases at the rate of 1 mV/s, and the VPT value can be defined as the voltage level when the patient indicates that he or she first feels the vibration sense. The mean of three readings at each site was taken; the higher vibration unit value indicates poorer performance or greater sensory dysfunction.

### Neurological Symptom Score and Neurological Disability Score

We have extended our assessment by inquiring about symptoms related to neuropathy. We asked participants whether they were experiencing sensations such as burning, numbness, tingling, fatigue, cramping, and aching. If patients reported experiencing any of these symptoms, we further inquired about the specific location, whether it was in their feet, calves, or elsewhere. In addition, we investigated whether symptoms worsened during the day, at night, or both and how patients found relief, whether through activities such as walking, standing, or sitting/lying down.

We have employed the NSS, which is a widely used system that assesses neurological symptoms including burning, numbness, and tingling, as well as sensations of fatigue, cramps, and aches. If a participant exhibited positive symptoms, we asked detailed questions about the timing, location, and methods of symptom relief. The NSS score ranges from 0 to 9, with scores of 0–2 considered normal, 3–4 categorized as mild, 5–6 as moderate, and 7–9 as severe.^15^

To evaluate the impact of neuropathy on patients’ daily lives, we conducted clinical tests to assess ankle reflex, sensory impairment (including loss of vibration, proprioception, pain, temperature, and touch sensation), and the extent of sensory loss. The results were documented using the NDS system, which can have a maximum score of 10. The NDS is categorized as normal, mild, moderate, or severely disturbed based on scores ranging from 0 to 2, 3 to 5, 6 to 8, and 9 to 10, respectively.^15,16^

### Operational definition

Peripheral neuropathy is operationally defined based on three distinct criteria, and a positive diagnosis is established if any one of the following conditions is met: Peripheral neuropathy is diagnosed by employing the NSS and NDS questionnaires. In accordance with Young’s criteria15, a positive diagnosis of peripheral neuropathy is confirmed when the combined score of the NSS and NDS exceeds 10.

The sensitivity and specificity for NDS+NSS >10 in detecting early DPN were 71 and 90%15. Clinical studies have provided strong validation for the utilization of VPT in the diagnosis of neuropathy, as evidenced by its sensitivity and specificity of 80 and 98%, respectively.^9^ This is additionally supported by extensive prospective epidemiological studies which demonstrate that a VPT exceeding 25 mV exhibited a sensitivity of 83% and a specificity of 63%.^8^

Patients scoring 10 or below are categorized as having normal results. Peripheral neuropathy is diagnosed based on clinical examination findings, which include the presence of any of the following physical signs in one or both lower limbs: diminished or absent ankle reflex; reduced vibration sensation as determined by a 128-Hz tuning fork; impaired pinprick sensation, indicating a reduced ability to perceive pain; and altered temperature discrimination.

The assessments on ankle reflex and sensory impairment were conducted by two trained doctors and validated by experienced researchers who consist of family medicine specialists and neurologists.

Peripheral neuropathy diagnosis can also be established through the utilization of the biothesiometer test.^8^ Specific cutoff values are employed to determine the presence of peripheral neuropathy: A value below 15 V is indicative of the absence of peripheral neuropathy; a range of 15–24.9 V suggests mild peripheral neuropathy; and a value of 25 V or more indicates significant peripheral neuropathy.

When biothesiometer readings indicate mild to severe peripheral neuropathy, this condition is categorized as “peripheral neuropathy.” Conversely, when biothesiometer readings fall within the typical range, they are classified as “no peripheral neuropathy.”^8^ In addition, when using biothesiometry to diagnose peripheral neuropathy,^14^ the following threshold values for vibration perception and corresponding neuropathy categories apply based on age: for individuals over the age of 50 years: 1–15 V: Normal; 16–20 V: Mild; 21–25 V: Moderate; 26–50 V: Severe. Whereas for the individuals aged 50 years and below: 1–10 V: Normal; 11–15 V: Mild; 16–20 V: Moderate; 21–50 V: Severe. Neurological disorders are defined as Guillain-Barre syndrome and chronic inflammatory demyelinating polyneuropathy.

### Statistical analysis

Statistical analyses were performed using version 26.0 of the Statistical Package for the Social Sciences.^18^ Descriptive statistics were utilized to compute the mean and standard deviation (SD) or median and interquartile range (IQR) for the baseline characteristics of the study participants. The association between categorical data was examined using either chi-square test. The association between continuous data and categorical data was examined using independent Student’s t-test or one-way ANOVA test. The level of statistical significance was set at a p-value <0.05.

### Ethical approval

We obtained ethical clearance from the Medical Research Ethics Committee (MREC), Ministry of Health Malaysia, under reference NMRR-20-971-54860, and from the Ethics Committee for Research Involving Human Subjects, Universiti Putra Malaysia, with reference number JKEUPM-2020-367. A written consent was obtained from all respondents prior to data collection.

## Results

In this study, a total of 1283 participants were enrolled with an average age of 40.6 ± 12.9 years. The majority of the participants were of Chinese ethnicity (54.1%) and 43.4% had completed tertiary education. A significant proportion of the participants did not consume alcohol (80.6%), were non-smokers (83.5%), and were non-vegetarian (97.6%). Among the participants, the percentage of individuals with hypertension (21.8%) was higher than those with diabetes (12.9%). In addition, a small proportion of participants reported having underlying neurological problems (3.2%) and 7.3% had a family history of neurological problems (Table 1).

Age is a significant factor, with increasing neuropathy severity in older participants. Males were more likely to exhibit severe peripheral neuropathy compared with females. In addition, the Indian ethnic group displayed higher severity levels compared with others.

In the case of the NSS, age was strongly associated with symptom severity, with older individuals experiencing more severe symptoms. However, gender and ethnicity did not significantly associate NSS scores. For the NDS, age remained a critical factor, indicating that older individuals had more severe disability. Gender did not show a substantial association with NDS scores, while ethnicity played a notable role in determining neuropathy severity. In clinical examination findings, age was again a significant determinant, with increasing abnormalities in older individuals. Gender did not show a significant association with clinical examination results, but ethnicity, specifically the Indian demographic, exhibited a higher prevalence of abnormalities compared with others. In summary, age consistently influences neuropathy severity across all screening tools, while the impact of gender and ethnicity varies depending on the assessment method. These findings shed light on the intricate relationship between demographic factors and neuropathy assessment (Table 2).

Table 3 shows the effectiveness of biothesiometer test, NSS, NDS, and a combination of NSS and NDS in screening for peripheral neuropathy. Regarding the biothesiometer test, 182 participants had a positive test result for peripheral neuropathy based on clinical examination findings, while 109 had a negative result. Among those with a positive biothesiometer result, 53% actually had peripheral neuropathy, while, among those with a negative biothesiometer result, 88% did not have peripheral neuropathy based on clinical examination. The sensitivity of the biothesiometer test was 0.63, meaning that it correctly identified 63% of the participants who had peripheral neuropathy. The specificity was 0.84, indicating that the test correctly identified 84% of the participants who did not have peripheral neuropathy. The PPV was 0.53, meaning that the probability of having peripheral neuropathy given a positive test result was 53%. The NPV was 0.88, indicating that the probability of not having peripheral neuropathy given a negative test result was 88%.

Combining the NSS and NDS with a threshold of greater than 10 improved sensitivity compared with using the individual scores. The combined tool had high specificity and a high PPV, suggesting that it could be a reliable indicator of peripheral neuropathy when both scores are elevated. The sensitivity of this test was very low at 0.11, meaning that it only correctly identified 11% of the participants who had peripheral neuropathy. However, the specificity was very high at 1.00, indicating that the test correctly identified all of the participants who did not have peripheral neuropathy. The PPV was 1.00, meaning that the probability of having peripheral neuropathy given a positive test result was 100%. The NPV was 0.79, indicating that the probability of not having peripheral neuropathy given a negative test result was 79% (Table 3).

The sensitivity and specificity findings of this study apply specifically to the Diabetic Foot Care Model: Vibrometer-VPT model 1. While there may be a degree of variability from model to model, we did not cross-reference the data with other models. Consequently, the results must be interpreted with caution.

## Discussion

The study was on the accuracy of the NSS, the NDS, and the biothesiometer against clinical examination findings for the identification of peripheral neuropathy among adults in retail pharmacies in Selangor, Malaysia. Peripheral neuropathy diagnosis based on clinical examination findings was used as the standard test which reflects the real-life situation when compared with NDS, NSS, and the biothesiometer test.

The results of the study showed that age was a significant factor in determining neuropathy severity across all screening tools. Older participants exhibited more severe neuropathy symptoms, disability, and clinical examination abnormalities. Males were more likely to exhibit severe peripheral neuropathy compared with females, and the Indian ethnic group displayed higher severity levels compared with others in the biothesiometry test. However, gender and ethnicity had varying impacts on neuropathy severity depending on the assessment method. The findings of this study are consistent with previous research that has shown age to be a significant factor in determining neuropathy severity.^19^ The biothesiometry test, in particular, has been shown to be a reliable and sensitive tool for detecting neuropathy severity in older individuals.^20^ The NSS and NDS tests have also been shown to be useful in assessing neuropathy severity, with age being a critical factor in determining symptom severity and disability.^8^ However, the impact of gender and ethnicity on neuropathy severity has been less consistent across different assessment methods.^21,22^

The biothesiometer test showed moderate sensitivity (0.64) and specificity (0.84) in diagnosing peripheral neuropathy, making it a reasonable tool for identifying peripheral neuropathy. The test’s NPV was particularly high, suggesting its effectiveness in ruling out the condition. These findings are consistent with previous studies that have reported similar sensitivity and specificity values for the biothesiometer test with a cut-off value of ≥20.5 V.^8,17^ The sensitivity and specificity increase to 82% and 70% when the cut-off value raises to ≥24.5 V. However, the PPV of the biothesiometer test was low, indicating that a positive test result may not necessarily indicate the presence of peripheral neuropathy. Therefore, further evaluation and diagnostic testing may be required in cases of a positive biothesiometer test result. This may be contributed by the amount of pressure applied to the probe, psychological factors, and choice of limb site.^23^ For example, biothesiometer is the choice to detect large nerve fiber-related^21^ peripheral neuropathy in the lower extremity.^24^

The combination of the NSS and NDS, with a score greater than 10 indicating a positive test result for peripheral neuropathy, showed high specificity but low sensitivity. While the high specificity indicates that this test is useful in ruling out peripheral neuropathy, the low sensitivity may result in false negatives and missed diagnoses such as subclinical neuropathy.^25^ Subclinical neuropathy presents in 20% of diabetic patients.^26^ This highlights the importance of using multiple screening tools and diagnostic tests in the diagnosis of peripheral neuropathy.

On the basis of the symptom profiles and risk factors of patients suspected of having peripheral neuropathy, the results may assist clinicians in selecting appropriate diagnostic procedures. For example, the biothesiometer’s suitability for preliminary quantitative sensory evaluation is supported by its comparatively high sensitivity and specificity. Table 4 summarizes the sensitivity, specificity, and problems encountered by different diagnostic tools in detecting peripheral neuropathy. Nevertheless, our findings unequivocally illustrate the necessity for composite screening methodologies that incorporate various instruments, such as the NSS and NDS questionnaires. Suppressing alternative diagnostic methods in favor of a single isolated modality may result in overlooked diagnoses attributable to the modest accuracy of individual tests.

When assessing peripheral neuropathy, clinicians should utilize biothesiometry, sensory/motor symptom tests, and clinical examination findings such as reflexes/sensation, according to this key practice implication. In contrast to singular approaches, this holistic strategy exhibits a higher propensity for accurately ascertaining the presence and severity of disease. Timely intervention, including symptomatic treatment, glycemic control, and lifestyle modification, can be enhanced by a prompt and accurate diagnosis, which additionally enables the prevention of neuropathy progression.

In summary, the results of this study suggest that the biothesiometer test, NSS, and NDS are valuable screening tools for peripheral neuropathy, but further evaluation and diagnostic testing may be necessary for positive results. Clinical examination remains crucial in diagnosis. A comprehensive approach combining multiple screening tools and tests is recommended to improve diagnostic accuracy and prevent complications.

### Strengths and limitations

The reported sensitivity, specificity, PPV, and NPV of the screening tools provide clinically relevant information. For example, the biothesiometer’s moderate sensitivity implies potential missed cases, highlighting the need for thorough assessment. Convenient sampling from pharmacies may introduce selection bias, but in view of the large and diverse sample size mitigates concerns about generalizability. Future research should prioritize population-based random sampling.

### Study Recommendation

These findings have important implications for diagnosing and managing peripheral neuropathy. Age, gender, and ethnicity should be considered in assessment methods. Further research is needed to establish standardized diagnostic criteria and evaluate tool accuracy across populations. The biothesiometer’s equivalent sensitivity and specificity, user-friendliness, portability, and objective results make it suitable for community use, potentially aiding in peripheral neuropathy awareness at the community level.

## Conclusion

This study provides valuable insights into the intricate relationship between demographic factors and neuropathy assessment. Age consistently influences neuropathy severity across all screening tools, while the impact of gender and ethnicity varies depending on the assessment method. These findings can help clinicians better understand the complex nature of peripheral neuropathy and develop more accurate and effective diagnostic and management strategies.

## Authors’ Contributions

Conceptualization, Funding acquisition, Methodology, Resources, Supervision, Visualization, Writing – original draft, Writing – review & editing – CM. Conceptualization, Methodology, Project administration, Software, Visualization, Writing – original draft, Writing – review & editing – KL. Conceptualization, Methodology, Writing – review & editing – AK. Conceptualization, Data collection, Methodology, Writing – review & editing – ND. Conceptualization, Data collection, Supervision, Writing – review & editing – AC. Conceptualization, Data collection, Resources, Supervision, Writing – review & editing – FH. Conceptualization, Data collection, Validation, Writing – review & editing – WS. Conceptualization, Data collection, Validation, Writing – review & editing – WL. Project administration, Writing – original draft, Writing – review & editing – LJ. Project administration, Writing – original draft, Writing – review & editing – TH. Writing – review & editing – VR.

## Acknowledgment

This research was funded by Procter & Gamble (M) Sdn. Bhd (Vote ID: 6380068). The funder had no role in study design, data collection and analysis, the decision to publish, or the preparation of the manuscript.

## Conflicts of Interest

The authors declared that there are no conflicts of interest.

## Figures and Tables

**Table 1.** Socio-demographics, lifestyle, medical history, family history, and h/o diabetes-related complications (*n* = 1283).

**Variables**	***n*(%)**	**Mean (SD)**
Age, (years)		40.6 (12.9)
Gender		
Male	648 (50.5)	
Female	635 (49.5)	
Ethnicity		
Malay	372 (29.0)	
Chinese	694 (54.1)	
Indian	161 (12.5)	
Others	56 (4.4)	
Education		
None	39 (3.0)	
Primary school	126 (9.8)	
Secondary school	446 (34.8)	
Pre-university	115 (9.0)	
Tertiary level	557 (43.4)	
Personal monthly income, in Ringgit Malaysia		4246.2 (4403.3)
Drinking alcohol	249 (19.4)	
Smoker	212 (16.5)	
Vegetarian	31 (2.4)	
Hypertension	280 (21.8)	
Diabetes	166 (12.9)	
Have neurological disorders	41 (3.2)	
Family history of neurological disorders	94 (7.3)	
Complications among those with diabetes		
Stroke	11 (6.6)	
Ischemic heart disease	26 (15.7)	
Renal disease	15 (9.0)	
Retinopathy	43 (25.9)	
Foot ulcer	6 (3.6)	

**Table 2.** Comparison of severity of peripheral neuropathy and socio-demographic data using chi-square. Independent Student’s t-test or one-way ANOVA test (*n* = 1283).

**Screening tests**	**Severity category, *n*(%)**				***p*-value**
**Biothesiometer test**	Normal, 943 (73.5)	Mild, 114 (11.2)	Moderate, 60 (4.7)	Severe, 136 (10.6)	
Age, mean ± SD	41 ± 14	54 ± 13	60 ± 11	65 ± 12	<0.001
Gender					
Female	495 (78.0)	72 (11.3)	25 (3.9)	43 (6.8)	<0.001
Male	448 (69.1)	72 (11.1)	35 (5.4)	93 (14.4)	
Ethnicity					
Malays	302 (81.2)	32 (8.6)	10 (2.7)	28 (7.5)	0.023
Chinese	492 (70.9)	81 (11.7)	39 (5.6)	82 (11.8)	
Indian	111 (68.9)	21(13.0)	8(5.0)	21(13.0)	
Others	38 (67.9)	10 (17.9)	3(5.40)	5 (8.9)	
**Neuropathy symptom scale**	Normal, 917 (71.5)	Mild, 173 (13.5)	Moderate, 150 (11.7)	Severe, 43 (3.4)	
Age, mean ± SD	43 ± 16	54 ± 16	52 ± 16	55 ± 15	<0.001
Gender					
Female	440 (69.3)	92 (14.5)	80 (12.6)	23 (3.6)	0.401
Male	477 (73.6)	81(12.5)	70(10.8)	20(3.1)	
Ethnicity					
Malays	268 (72.0)	48 (12.9)	42 (11.3)	14(3.8)	0.943
Chinese	498 (71.8)	94 (13.5)	79 (11.4)	23 (3.3)	
Indian	110 (68.3)	24 (14.9)	21 (13.0)	6 (3.7)	
Others	41 (73.2)	7 (12.5)	8 (14.3)	0 (0.0)	
**Neuropathy disability scale**	Normal, 1140 (88.9)	Mild, 88 (6.9)	Moderate, 51 (4.0)	Severe, 4 (0.3)	
Age, mean ± SD	43 ± 16	58 ± 12	70 ± 12	69 ± 5	<0.001
Gender					
Female	578 (91.0)	37 (5.8)	18 (2.8)	2 (0.3)	0.081
Male	562 (86.7)	51 (7.9)	33 (5.1)	2 (0.3)	
Ethnicity					
Malays	346 (93.0)	19 (5.1)	6 (1.6)	1 (0.3)	0.011
Chinese	609 (87.8)	48 (6.9)	35 (5.0)	2 (0.3)	
Indian	136 (84.5)	15 (9.3)	10 (6.2)	0 (0)	
Others	49 (87.5)	6 (10.7)	0 (0)	1 (1.8)	
**Clinical examination finding**	Normal, 882 (77.3)		Abnormal, 291 (22.7)		
Age, mean ± SD	42 ± 15		59 ± 15		<0.001
Gender					
Female	499 (78.6)		136 (21.4)		0.285
Male	493 (76.1)		155 (23.9)		
Ethnicity					
Malays	316 (84.9)		56 (15.10)		<0.001
Chinese	518 (74.6)		176 (25.4)		
Indian	114 (70.8)		47 (29.2)		
Others	44 (78.6)		12 (21.4)		

A positive clinical examination is determined by the presence of any physical examination findings on either one or both lower limbs, as described below: reduced or absence of ankle reflex or reduced vibration sensation using a 128-Hz tuning fork or reduced pinprick sensation (i.e., reduced ability to feel pain) or reduced temperature discrimination. SD, standard deviation.

**Table 3.** Sensitivity, specificity, positive predictive value, and negative predictive values of screening tools based on biothesiometer, neuropathy symptom score, and neuropathy disability score (*n* = 1283).

**Name of the tool**	**Result of the screening tools**	*Have peripheral neuropathy	**No peripheral neuropathy**	**Total, *n***
Biothesiometer test	Positive, *n*	182	158	340
	Negative, *n*	109	834	943
	Total, *n*	291	992	
	Sensitivity	0.63		
	Specificity	0.84		
	Positive predictive value	0.53		
	Negative predictive value	0.88		
		*Have peripheral neuropathy	No peripheral neuropathy	Total, *n*
NSS	Positive, *n*	27	16	43
	Negative, *n*	965	275	1240
	Total, *n*	992	291	
	Sensitivity	0.03		
	Specificity	0.95		
	Positive predictive value	0.63		
	Negative predictive value	0.22		
		*Have peripheral neuropathy	No peripheral neuropathy	Total, *n*
NDS	Positive, *n*	0	4	4
	Negative, *n*	992	287	1279
	Total, *n*	992	291	
	Sensitivity	0		
	Specificity	0.98		
	Positive predictive value	0		
	Negative predictive value	0.22		
		*Have peripheral neuropathy	No peripheral neuropathy	Total, *n*
NSS+NDS>10	Positive, *n*	32	0	32
	Negative, *n*	259	992	1251
	Total, *n*	291	992	
	Sensitivity	0.11		
	Specificity	1		
	Positive predictive value	1		
	Negative predictive value	0.79		

* Have peripheral neuropathy based on clinical examination findings which include the presence of any of the following physical signs in one or both lower limbs: diminished or absent ankle reflex or reduced vibration sensation as determined by a 128-Hz tuning fork or impaired pinprick sensation, indicating a reduced ability to perceive pain or altered temperature discrimination. NSS, Neurological Symptom Score; NDS, Neurological Disability Score.

**Table 4.** Summary of the sensitivity, specificity, and problem encountered by different diagnostic tools in detecting peripheral neuropathy.

**Tools**	**Sensitivity (%)**	**Specificity (%)**	**Comments on strength or limitations**
Biothesiometer	80–83%^9^ 63% (this study)	63–98%^9^ 84% (this study)	Objective quantification of vibration perception, easyand quick to perform.
NSS + NDS >10	71%^12^ 11% (this study)	90%^12^ 100% (this study)	Evaluates symptoms, prone to reporting bias, simple, low-cost
MNSI <2	96.8% (this study)	85.7% (this study)	Evaluates both symptoms and signs, composite measure makes, validatedscreening algorithm interpretation complex

NSS, Neurological Symptom Score; NDS, Neurological Disability Score; MNSI, The Michigan Neuropathy Screening Instrument.
